# Exosomal CagA from *Helicobacter pylori* aggravates intestinal epithelium barrier dysfunction in chronic colitis by facilitating Claudin-2 expression

**DOI:** 10.1186/s13099-022-00486-0

**Published:** 2022-03-24

**Authors:** Yinjie Guo, Canxia Xu, Renjie Gong, Tingzi Hu, Xue Zhang, Xiaoran Xie, Jingshu Chi, Huan Li, Xiujuan Xia, Xiaoming Liu

**Affiliations:** 1 Department of Gastroenterology, Third Xiangya Hospital of Central South University, 138 Tongzipo Road, Changsha, 410013 China; 2 Department of Ophthalmology, The Second Xiangya Hospital, Central South University, Changsha, 410008 China; 3 Hunan Key Laboratory of Nonresolving Inflammation and Cancer, Changsha, 410013 China

**Keywords:** CagA, Exosome, Colitis, Tight junction, Claudin-2, CDX2

## Abstract

**Background:**

The chronic infection with *Helicobacter pylori* (*H. pylori*), especially cytotoxin-associated gene A-positive (CagA^+^) strains, has been associated with various extragastric disorders. Evaluating the potential impacts of virulence factor CagA on intestine may provide a better understanding of *H. pylori* pathogenesis such as colitis. The intestinal mucosal barrier is essential for maintaining its integrity and functions. However, how persistent CagA^+^ *H. pylori* colonization influences barrier disruption and thereby affects chronic colitis is not fully understood.

**Results:**

Chronic colitis models of CagA^+^ *H. pylori*-colonized mice treated with 2% Dextran sulphate sodium (DSS) were established to assess the disease activity and pertinent expression of tight junction proteins closely related to mucosal integrity. The aggravating effect of CagA^+^ *H. pylori* infection on DSS-induced chronic colitis was confirmed in mouse models. In addition, augmented Claudin-2 expression was detected in CagA^+^ *H. pylori* infection conditions and selected for mechanistic analysis. Next, GES-1 human gastric epithelial cells were cultured with CagA^+^ *H. pylori* or a recombinant CagA protein, and exosomes isolated from conditioned media were then identified. We assessed the Claudin-2 levels after exposure to CagA^+^ exosomes, CagA^−^ exosomes, and IFN-γ incubation, revealing that CagA^+^ *H. pylori* compromised the colonic mucosal barrier and facilitated IFN-γ-induced intestinal epithelial destruction through CagA-containing exosome-mediated mechanisms. Specifically, CagA upregulated Claudin-2 expression at the transcriptional level via a CDX2-dependent mechanism to slow the restoration of wounded mucosa in colitis in vitro.

**Conclusions:**

These data suggest that exosomes containing CagA facilitate CDX2-dependent Claudin-2 maintenance. The exosome-dependent mechanisms of CagA^+^ *H. pylori* infection are indispensable for damaging the mucosal barrier integrity in chronic colitis, which may provide a new idea for inflammatory bowel disease (IBD) treatment.

**Supplementary Information:**

The online version contains supplementary material available at 10.1186/s13099-022-00486-0.

## Introduction

*Helicobacter pylori* (*H. pylori*), a Gram-negative microaerophilic bacterium in the stomach, is one of the most ubiquitous pathogens as it colonizes more than half of the world’s population [1]. *H. pylori* infection is closely associated with several gastric diseases, including gastritis, peptic ulcer disease, and gastric cancer [2]. Among the reported pathogenic factors of *H. pylori*, cytotoxin-associated gene A protein (CagA) has been the focus of attention. CagA is a specific virulence factor protein of *H. pylori* that enters host cells through the type IV secretion system (T4SS). Once inside the cell, CagA triggers a variety of signal transduction pathways, induces changes in cell morphology, and increases the risk of gastric diseases [3, 4]. Moreover, increasing evidences show that CagA^+^ *H. pylori* strains other than CagA^−^ *H. pylori* are pathogens closely related to diseases outside the stomach [5, 6]. Deposited in renal tubules, CagA brings about strongly mucosal immune response in IgA nephropathy [7]. CagA^+^ *H. pylori* strains induce premature senescence of extragastric cells, which may contribute to chronic skin diseases [8]. CagA seropositivity are highly correlated with sarcopenia and low muscle quantity [9]. In the context of CagA’s contribution to extragastric diseases, a large proportion of conundrums such as inflammatory bowel disease (IBD) remain to be fully elucidated.

Among the defensive mechanisms of the intestinal mucosa, an impaired epithelial barrier is the main pathological factor that causes colitis, and it contributes to increased permeability by allowing increased antigen penetration thereby initiating and propagating the dysregulated response [10–13]. The normal intestinal barrier consists of an intact layer of epithelial cells, which are connected by a system of tight junction (TJ) strands [14]. TJs are composed of the occludin and claudin family members, which are transmembrane proteins, as well as TJ-associated proteins, such as Claudin-2 and ZO-1 [15, 16]. Claudin-2 has been shown to induce cation-selective channels in the tight junctions of epithelial cells, and its expression is increased in IBD, leading to diarrhea through the leakage flux mechanism [17, 18]. It was recently reported that Claudin-2 degradation enhances TJ barrier function in intestinal epithelial cells [19]. Specifically, Claudin-2 expression is positively associated with inflammatory activity in patients with IBD, and plays multiple roles, such as targeting the vitamin D receptor, in IBD pathogenesis [20, 21]. Another TJ protein, ZO-1, connects occludins and claudins to cytoskeletal actin, and the loss of ZO-1 leads to increased intestinal permeability [16]. ZO-1 preserves the intestinal barrier, and decreased intestinal inflammation has been observed [22, 23]. To the best of our knowledge, the potential implications of TJ dysregulation in IBD pathogenesis remain elusive.

Certain clinical studies and systematic reviews have indicated the association between *H. pylori* exposure and reduced IBD risk [24–26]. The clinically observed inverse association between *H. pylori* and IBD could be partly explained by the fact that *H. pylori* might exert an immunomodulatory effect in IBD since its DNA inhibits proinflammatory cytokine production of dendritic cells, equilibrates Th17/Treg and Breg cell responses, and shifts macrophages toward M2 anti-inflammatory environments [27–30]. Intriguingly, when considering the exact pathogenic or protective effects of CagA on IBD or colitis at the molecular levels, the conclusions are lacking. For example, dextran sulphate sodium (DSS)-induced colitis was prominently deteriorated in CagA-transgenic mice [31]. The biological basis for this effect regarding mucosal barrier integrity remains unclear.

Exosomes are extracellular vesicles derived from multivesicular bodies and are secreted by healthy cells, cancer cells, and host cells [32]. During infection, exosomes released from host cells can convey pathogenic components [33]. Exosomes are key regulators of cellular physiological functions and pathogenesis [34]. A recent study reported that exosomes containing CagA were released from *H. pylori* CagA-expressing cells and could be detected in blood circulation [35]. Moreover, exosomes released from *H. pylori*-infected host gastric epithelial cells can enter the bloodstream and aortic plaques, promote the formation of macrophage-derived foam cells, and accelerate the progression of atherosclerosis [36, 37]. Thus, exosome-carried CagA protein may be involved in the development of a variety of extragastric diseases including IBD.

To elucidate how *H. pylori* infection could affect IBD-associated chronic colitis via TJs, mouse models were established, and in vitro experiments were conducted in the present study. CagA^+^ *H. pylori* infection was used to test the hypothesis that exosomal CagA from *H. pylori* compromised the barrier integrity of the intestinal epithelium in colitis by facilitating Claudin-2 expression.

## Results

### CagA^+^***H. pylori*** infection increases Claudin-2 expression and aggravates DSS-induced chronic colitis in C57BL/6 mice

**Fig. 1:**
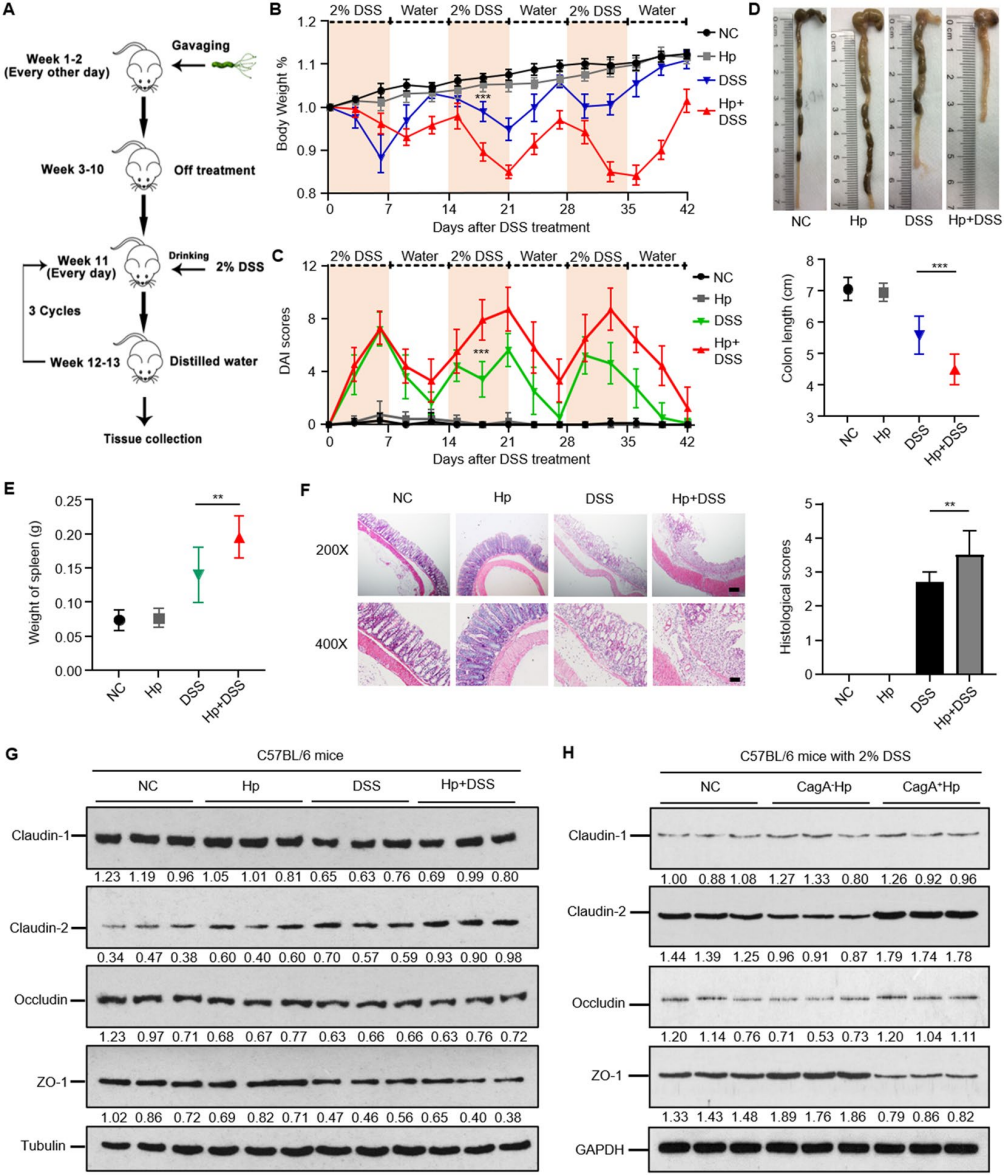
CagA^+^ *H. pylori* infection exacerbates DSS-induced chronic colitis in mice. **A** Schematic depiction of the experimental design for *H. pylori* (CagA^+^ strain) inoculation followed by chronic colitis models administrated 2% DSS. **B**, **C** Body weights (**B**) and disease activity index (DAI) changes (**C**) in different groups. Data from the second and third DSS-treatment cycles (7 day 2% DSS and 7 day diluted water) showed that *H. pylori-*infected mice had significantly less body weight loss and disease manifestation than control mice with no *H. pylori* infection in DSS-treated conditions. ***p < 0.001, compared to DSS group. Student’s *t test* for body weights, and χ^2^ test for DAI scores. **D**, **E**, **F** Colon lengths (**D**), spleen weights (**E**), microscopic appearance and H&E histological sections of the colon **F** in each group. Scale bars, 200 µm (200 ×) and 100 µm (400 ×). **p < 0.01, ***p < 0.001. Student’s *t test* was used for colon length and spleen weights, and the χ^2^ test was used for histological scores. **G** The relative expression of tight junction proteins (TJs) in the colon of *H. pylori* and chronic colitis mice. The augmented Claudin-2 was observed in the *H. pylori* + DSS group in comparison with the DSS group. **H** The TJs protein expression of chronic colitis mice subgroups without *H. pylori*, with CagA^−^ *H. pylori* infection, and with CagA^+^ *H. pylori* infection. Claudin-2 was significantly upregulated in CagA^+^ *H. pylori*-infected group compared with CagA^−^ *H. pylori* mice. All data were presented as means ± SD (n = 10)

To determine the effect of *H. pylori* on the severity of DSS-induced colitis, we first established a *H. pylori*-infected C57BL/6 mouse model under chronic inflammation conditions (Fig. 1A). Histopathological analysis of the mouse gastric tissues of the mice after 8 weeks of *H. pylori* (CagA^+^ strain) infection showed positive Giemsa and silver staining for *H. pylori* in all the tested animals, confirming that the mouse model of *H. pylori* infection was successful (Additional file 3: Figure S1). We then compared and analyzed the body weight and disease activity index (DAI) of mice with and without *H. pylori* infection every three days after 2% DSS administration. The anatomical and histopathological features of the colon and spleen in each group were also evaluated. As expected, the DSS group showed significant weight loss and higher DAI scores. In comparison with the normal control (NC) group, the mice with *H. pylori* infection alone (Hp group) displayed no significant changes in weight or DAI scores. However, the mice pretreated with *H. pylori* infection exhibited more weight loss and manifestations than the DSS-treated group, and the more intriguing finding was that these damaging effects of *H. pylori* colonization on chronic colitis were obviously detected in the second and third 2% DSS cycles (Fig. 1B, C).

All the mice with DSS-induced colitis showed varying degrees of shorter intestine, larger spleen weight, and loss of crypt and goblet cells with distinct mucosal and submucosal infiltration of inflammatory cells, while *H. pylori*-pretreated mice showed significantly distinct anatomical and histopathological abnormities (Fig. 1D, E, and F). The above data suggest a possible injuring role of *H. pylori* in enhancing DSS-induced chronic colitis, which is associated with incomplete regulation of the mucosal epithelium by TJs. Western blot analysis of the expression of potential TJs was performed in the control and experimental groups. Claudin-2 level was gradually increased in Hp and DSS group, reaching the peak in Hp + DSS groups, as expected, while claudin-2 levels gradually increased in the Hp and DSS groups, peaking in the Hp + DSS group. ZO-1 expression was substantially downregulated in the DSS groups, as expected, while the increasing tendency of ZO-1 in Hp + DSS group is not obvious. No changes in occludin were observed among the abovementioned groups (Fig. 1G).

The trend of Claudin-2 expression might explain how *H. pylori* exerts its aggravating effect in DSS-induced chronic colitis. Since the utilized bacterial in previous study was CagA^+^*H.pylori* strain, we tried our best to address if CagA, the strongest virulent factor for *H. pylori*, was involved in varied barrier function in chronic colitis status. Both CagA^+^ *H.pylori* and CagA^−^ *H.pylori* were inoculated into mice followed by 2% DSS induced colitis conditions (CagA negative status was identified in Additional file 4: Figure S2). The colon histological sections, colon lengths, and spleen weight verified that aggravated inflammation and intestinal injury were detected in CagA^+^*H.pylori* group compared to CagA^−^ *H.pylori* group (Additional file 5: Figure S3A, B, and C). As for TJs tendency, claudin-2 levels in CagA^+^*H.pylori* group was markedly increased in comparison with CagA^−^ *H.pylori* group. Intriguingly, CagA^−^ *H.pylori* was not responsible for upregulated claudin-2 compared to chronic colitis mice subgroup without *H. pylori* infection (Fig. 1H). Collectively, CagA^+^ *H. pylori* infection increases Claudin-2 expression and aggravates DSS-induced chronic colitis.

### CagA-containing exosomes upregulate Claudin-2 expression to compromise the intestinal mucosal barrier integrity in vitro

**Fig. 2:**
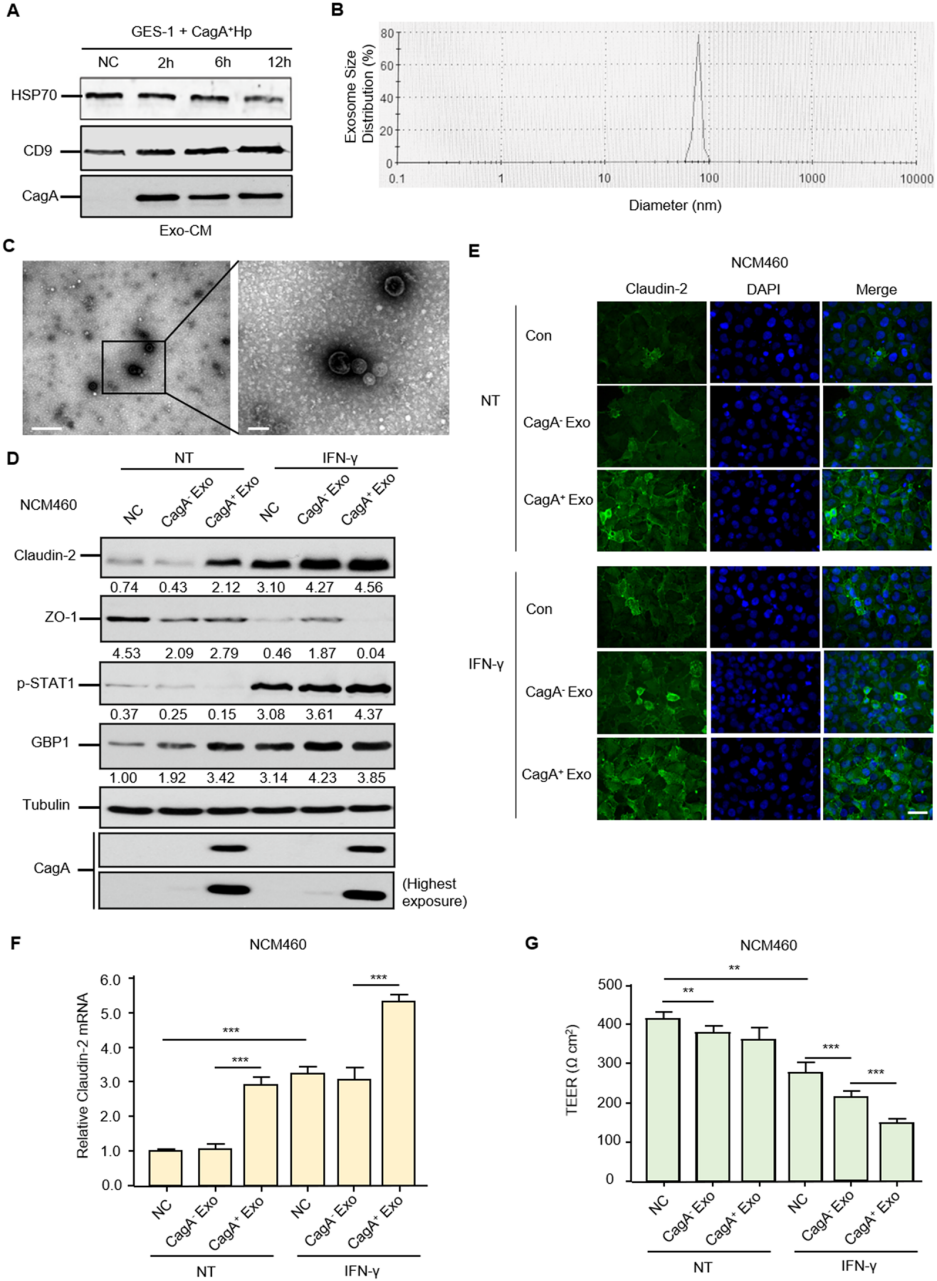
Exosomes from GES-1 human gastric epithelial cells cultured with CagA^+^ *H. pylori* significantly disrupt the intestinal mucosal barrier meanwhile and upregulate Claudin-2 in vitro. **A** Western blotting analysis confirmed exosomes isolated from GES-1 and CagA^+^ *H. pylori* coculture media via characteristic biomarkers HSP70 and CD9. The presence of CagA within the exosome was also shown. Exo-CM, exosome derived from conditioned medium. **B**, **C** Features of exosomes in terms of size distribution (**B**) and morphology (**C**) on transmission electron microscopy. Scale bar, 0.5 µm and 200 nm. **D** Western blotting verified the augmentation of Claudin-2 proteins by CagA^+^ exosomes, while the CagA^+^ exosome group partially maintained Claudin-2 protein expression under IFN-γ conditions. p-STAT1 and GBP1 were employed as positive controls to show the inflammatory status in response to CagA^+^ exosomes, CagA^−^ exosomes, or IFN-γ. The p-STAT1 pathway was stringently dependent on IFN-γ, as expected, and the presence of a slight GBP1 band also showed a low degree of inflammation after the entry of CagA^+^ exosomes. The shrinkage of ZO-1, an acknowledged tight junction protein in both CagA^+^ exosomes and IFN-γ conditions, was observed as a positive control. **E** Cell immunostaining showed that CagA^+^ exosomes contributed to membrane Claudin-2 formation, whose increase was also observed in IFN-γ induced barrier function disorders. Scale bar, 20 µm. **F** RT–qPCR revealed the mRNA expression tendencies in different groups. **G** CagA^+^-containing exosomes accelerate the dysintegrity of the NCM460 cell monolayer under inflammatory factor IFN-γ conditions. The transepithelial electrical resistance (TEER) values of the cell monolayer were tested after CagA^+^ exosomes and IFN-γ 24 h. The data are expressed as the means ± SD of three independent experiments. ***p < 0.001, *t* test

To determine how CagA^+^ *H. pylori* infection could affect DSS-induced colitis, we hypothesized that CagA^+^ *H. pylori* exerted its function through CagA-containing exosome-mediated mechanisms. Typical exosomes were present in the conditioned media of GES-1 cells cultured with CagA^+^ *H. pylori* and were characterized by their specific biomarkers (HSP70, CD9, and CagA), size distribution, and morphology (Fig. 2A, B, and C). These western blotting and electron microscopy results confirm the uptake of exosomes by NCM460 cells, which is analogous to our previous immunofluorescence staining results that the unique *H. pylori* pathogenic factor CagA entered human GES-1 cells after they were incubated with CagA^+^ *H. pylori* [37].

Compared to CagA^−^ *H. pylori* strain, western blot and immunofluorescence showed that NCM460 cells exposed to exosomes from CagA^+^ *H. pylori* and GES-1 coculture conditions directly augmented Claudin-2 protein expression while depleting ZO-1 levels. Mucosal integrity and inflammatory condition were characterized before and after an inflammatory challenge with IFN-γ in NCM460 monolayers. IFN-γ treatment triggered Claudin-2 upregulation and disrupted ZO-1 formation, as expected, while the shrinking ZO-1 bands were detected both in the CagA^+^ exosome and CagA^−^ exosome groups. The protein expression of phospho-STAT1 and guanylate-binding protein 1 (GBP1) was concurrently examined by western blotting to confirm this specific and overall inflammatory response after IFN-γ and CagA^+^ exosome treatments (Fig. 2D and E).

We next confirmed the increased mRNA level of Claudin-2 after treatment with CagA^+^ exosomes, while IFN-γ had the similar effects on their transcription (Fig. 2F). These data collectively indicate that IFN-γ and CagA^+^ exosomes enhance Claudin-2 protein expression via transcriptional upregulation patterns. In the TEER experiment, treatment with GES-1-derived CagA-containing exosomes had a slight effect on the colon monolayer under normal conditions but significantly aggravated the collapse of mucosal integrity after cytokine IFN-γ interference (Fig. 2G). Notably, both CCK-8 and EdU assays confirmed that CagA-containing exosomes and IFN-γ stimulation did not cause changes in the proliferation or confluence of NCM460 cell monolayers (Additional file 6: Figure S4A and B). It is thus tempting to speculate that the alteration of Claudin-2 by CagA-containing exosomes and IFN-γ negatively correlates with the integrity of the intestinal mucosal barrier.

### CagA-associated Claudin-2 transcriptional upregulation is dependent on CDX2 activation

**Fig. 3:**
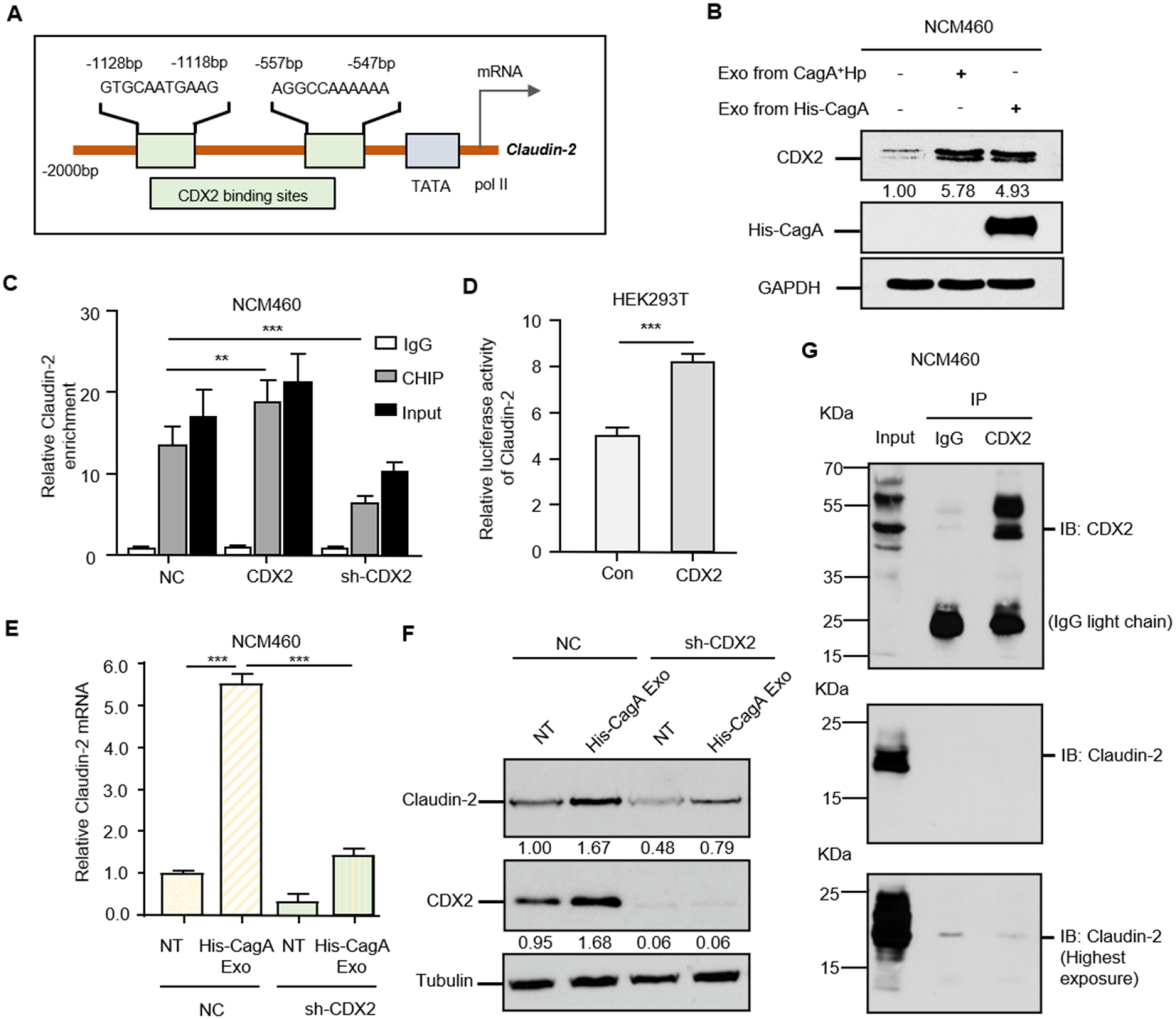
CagA activates CDX2 to transcriptionally upregulate Claudin-2 expression. **A** Schematic binding prediction of the transcription factor CDX2 in the *Claudin-2* gene promoter. The two positions and sequences with relatively high scores are indicated. **B** Western blotting confirmed the incremental CDX2 protein expression by CagA. In addition to exosomes from GES-1 gastric epithelial cells cultured with CagA^+^ *H. pylori*, recombinant His-CagA protein was utilized to perform CagA and GES-1 cell cocluture and subsequent exosome isolation. Both exosomes from CagA^+^ *H. pylori* infection and recombinant CagA protein incubation triggered CDX2 expression. The presence of CagA in exosomes was proven using an antibody against the His-tag. Notably, the more striking contrast of the CDX2 band was shown above at shorter exposure times due to its abundant endogenous expression in the colon. **C** Identification of the CDX2-Claudin-2 interaction using chromatin immunoprecipitation. CDX2 interferences were introduced into NCM460 cell. CDX2 protein was able to pull down Claudin-2 DNA fragments. IgG and Input were performed as controls. **D** The positive transcriptional regulation of Claudin-2 by CDX2 was confirmed by luciferase reporter assay. The ratio of firefly luciferase activity to Renilla activity was calculated to show the binding of CDX2 to gene promoter activities. Each experiment was performed in triplicate. **E**, **F** CDX2 depletion impeded CagA-associated Claudin-2 transcription and protein expression. Data are presented as the mean ± SD value from three biological replicates. **p < 0.01, ***p < 0.001, *t* test. **G** Coimmunoprecipitation precluded the protein interaction of CDX2 and Claudin-2. The equal CDX2 loading in the IP panel was preverified, and immunoblotting with Claudin-2 provided no binding of CDX2 and Claudin-2 even under the longest exposure

Based on the above results showing that Claudin-2 expression is transcriptionally regulated, we attempted to investigate whether potential transcription factors could act as decisive intermediates between exosome-carried CagA proteins and downstream TJs. The transcription factor CDX2 (Caudal related homeodomain transcription 2) was predicted to regulate Claudin-2 expression according to the Jasper database (Fig. 3A). In addition to exosomes from CagA^+^ *H. pylori* and GES-1 cell cocultures, human recombinant His-CagA protein was added to GES-1 cells, followed by exosome isolation. Significantly activated CDX2 was detected in exosomes from GES-1 cells treated with both CagA^+^ *H. pylori* and a foreign recombinant CagA protein, and the western blot results indicated that CagA, the main virulence factor of *H. pylori*, played a prominent role in CagA-containing exosome-mediated mechanisms (Fig. 3B).

The ChIP results showed that CDX2 could pull down Claudin-2 DNA fragments, and CDX2 overexpression/depletion resulted in significantly increased/decreased Claudin-2 signals (Fig. 3C). A luciferase reporter assay in a HEK293T cell model confirmed the binding of CDX2 in Claudin-2 promoters and a positive transcriptional pattern (Fig. 3D). Furthermore, CDX2 knockdown exhibited significantly greater inhibitory effects on Claudin-2 transcription and translation, even under conditions of CagA-containing exosome exposure, suggesting that Claudin-2 was mainly driven by CDX2 (Fig. 3E and F). Coimmunoprecipitation further precluded the protein interaction of nucleoprotein CDX2 and membrane protein Claudin-2 (Fig. 3G). Collectively, CDX2 could be activated by the CagA protein and is essential for transcriptional upregulation of Claudin-2.

### CDX2-dependent Claudin-2 activation impairs mucosal barrier integrity in chronic colitis in vitro

**Fig. 4:**
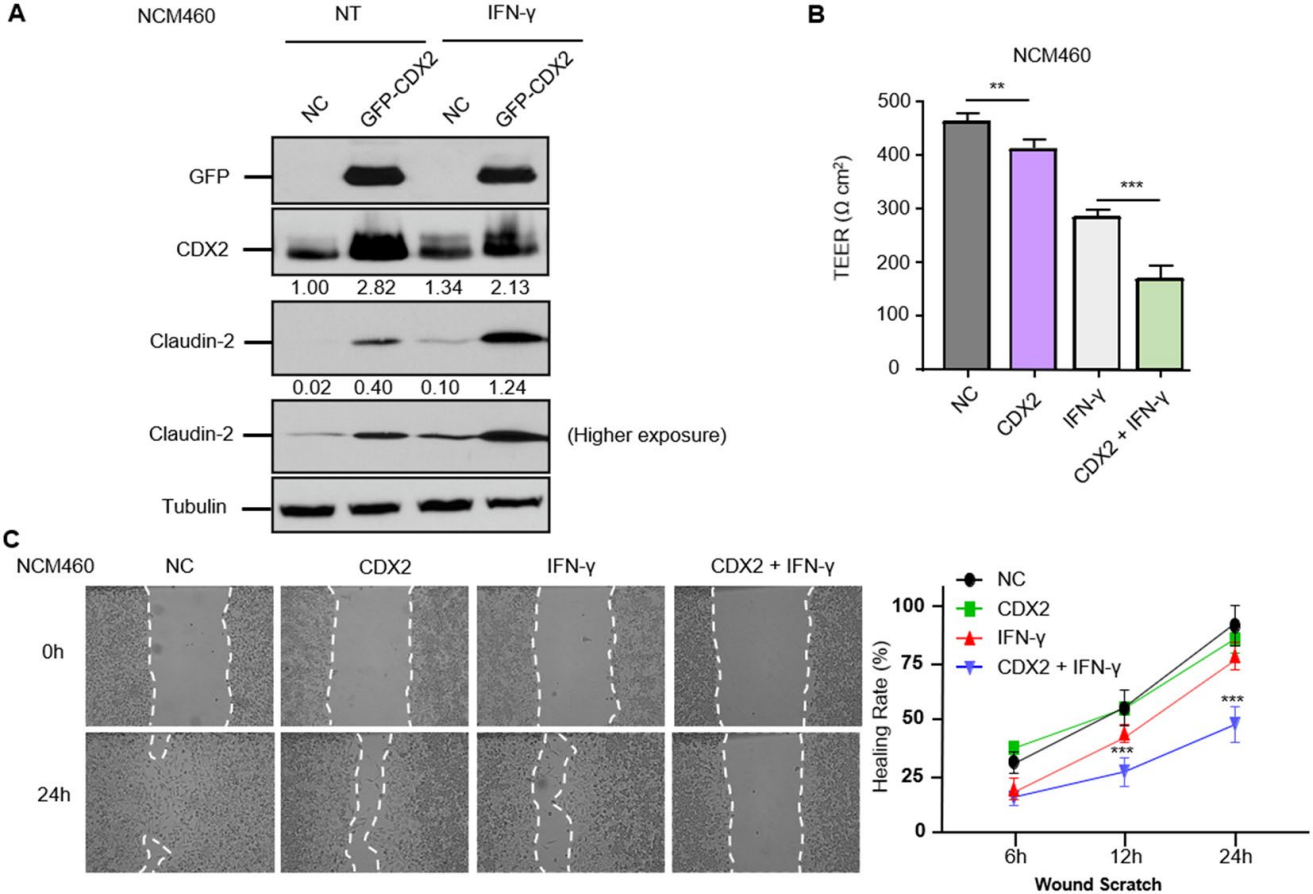
CDX2 is indispensable for Claudin-2 expression in mucosal barrier integrity and colonic epithelial restitution. **A** Overexpression of CDX2 upregulated Claudin-2 protein expression, and their abundances were detected even under IFN-γ incubation. The stable CDX2 overexpression model was employed using GFP-tagged vectors. **B** CDX2 partially impaired colonic mucosal barrier integrity in IFN-γ stimulation. The TEER values of the NCM460 cell monolayer under each condition were measured. **p < 0.01, ***p < 0.001, *t* test. **C** CDX2-associated Claudin-2 impeded colonic wound healing and partially strengthened the IFN-γ-induced mucosal deficiency. The wounds were recorded at 0 h, 6 h, 12 h, and ultimate 24 h. CDX2 + IFN-γ vs. IFN-γ, ***p < 0.001, *t* test

We then explored the relevant role of CDX2 in the mucosal barrier with respect to TJs, such as Claudin-2. When transfected with GFP-tagged CDX2, western blotting showed increased Claudin-2 expression. Treatment with IFN-γ induced colonic barrier damage, manifested as significantly upregulated expression of Claudin-2, which was also detected at higher levels in CDX2-overexpressing cells (Fig. 4A). These results were consistent with the TEER value tendency affected by IFN-γ and CDX2 (Fig. 4B). In further experiments, polarized monolayers of NCM460 vector control and CDX2 stable overexpressing cells were wounded and then incubated with IFN-γ. In untreated control and CDX2-overexpressing cells, approximately 89.1% and 87.2% of the wounded areas spontaneously healed at 24 h after wounding, indicating that CDX2 treatment slightly reduced migration but had no significant difference between the two groups. In control cells treated with IFN-γ, the healing rate was inhibited even further to 70.1%, whereas in CDX2-overexpressing monolayers treated with IFN-γ, 45.7% of the wounded area was healed (Fig. 4C). These data indicate that CDX2 positively regulates Claudin-2 expression and impairs mucosal barrier and wound healing functions in chronic colitis conditions in vitro.

## Discussion

Abundant evidence indicates that chronic *H. pylori* infection may contribute to the development of IBD. Therefore, it is essential to identify the mechanisms mediating disease initiation and progression for the development of prevention and treatment strategies for IBD. *H. pylori* mostly resides in the gastric epithelial layer, and a fraction of *H. pylori* can be found in the lamina propria of the submucosa close to gastric mucosa capillaries and postcapillary venules [38, 39]. However, no *H. pylori* has been detected in the blood of infected patients to directly affect colitis, and there is no evidence to define a causal relationship between this “remote infection” and intestinal mucosal integrity [40]. An exosome is a cell-derived vesicle that also plays an important role in cell-to-cell communication. The regulation of exosome-associated IBD has attracted increasing attention due to its potential exosome pathway function. Exosome-carried CagA protein can enter the circulation and transfer microbial virulence factors to sites far from the primary disease, which may cause seemingly unrelated clinical effects or symptoms [36]. This was also confirmed in our study, in which exosomal CagA was detected in the serum of mice infected with CagA^+^ *H. pylori*. We demonstrated that exosomal CagA from *H. pylori* disrupts the barrier function of intestinal epithelial cells in colitis by facilitating Claudin-2 expression.

Ideally, a *cagA* knockout strain derived from the corresponding CagA^+^ strain is better used for the control. However, we currently have some concerns on using the knockout mutants, since recent publications shown that knocking out gene affects the structure and function of *H. pylori,* such as retarded growth and unsuccessful bacterial infection, reduced bacterial adhesion and internalization in cell line model [41]. Even, in the multiple knockout mutants, CagA and VacA, the key virulence factors of *H. pylori*, were both substantially reduced [42]. Future studies are needed to explore whether there are significant differences among CagA wild-type, CagA depleted, and CagA mutated *H. pylori* strains in vivo and in vitro.

CDX2 is a transcription factor that has been shown to activate the Claudin-2 promoter in human intestinal epithelial cells and plays a crucial role in controlling the balance between proliferation and differentiation of intestinal epithelial cells [43–45]. An indirect pattern of ZO-1 regulation by CDX2 via the MEK/ERK pathway was also verified in porcine intestinal epithelium [46]. CDX2 expression is significantly decreased in patients with active UC, and it is involved in protection against DSS-induced colitis [47, 48]. However, to date, the regulation of mucosal inflammation by CDX2 as a triggering or compensative factor is rather unclear. Interestingly, a recent study indicated that CagA disrupts tight junctions by increasing the CDX2-dependent targeting of Claudin-2 in gastric carcinoma cells and causes significant changes in the morphology and activity of these cells [49]. In our experiment, exosomal CagA upregulated CDX2 expression and increased Claudin-2 expression to impair the intestinal mucosal barrier. Similarities in these results may be due to the infiltration of CagA exosomes into either the gastric or colonic epithelium irrespective of the different and complex intracellular environments. Moreover, increased CDX2 expression was associated with a more metastatic phenotype of gastric carcinoma cells [49], whereas CDX2 might act as a tumor suppressor during colorectal carcinogenesis [50]. These discrepancies led us to explore the fine regulation of CDX2 in both gastric and intestine epithelial cells as the CagA^+^ *H. pylori* related primary lesion and secondary effect, respectively.

In summary, we demonstrated for the first time that chronic infection with CagA^+^ *H. pylori* exacerbates DSS-induced UC-like colitis in mice. A series of in vitro experiments confirmed the exosomal CagA-associated upregulation of Claudin-2 expression by the activation of the transcription factor CDX2. The damaging role of CagA in mucosal barrier integrity was verified by a series of functional experiments under IFN-γ-stimulating conditions. Although it is not clear whether intestinal epithelial barrier dysfunction is a cause or a consequence of IBD, intestinal epithelial Claudin-2 can still be used as a target to impair the integrity of the IBD intestinal barrier, which provides a new idea for IBD treatment. Further research is needed to determine whether such a mechanism may also be involved in other extragastric diseases caused by *H. pylori* infection.

## Materials and methods

### *H. pylori* culture

CagA^+^ *H. pylori* and CagA^−^ *H. pylori* used in culture and mouse studies were isolated from gastric specimens of gastric ulcer patients during gastroscopy as described [37, 51]. In brief, bacteria were grown on Columbia blood agar plates supplemented with antibiotics (10 mg/L vancomycin, 5 mg/L cefsulodin, 5 mg/L amphotericin B, 5 mg/L trimethoprim, and 10% sheep blood (Thermo Scientific R54008, Waltham, MA)) at 37 °C under microaerophilic conditions (5% O_2_, 10% CO_2_, and 85% N_2_) for 3–4 days. After incubation for 3–4 days, the bacterial colonies were collected by scrape and then resuspended in PBS. The bacterial concentration was calculated based on an optical density (OD) of 1 × 10^8^ colony-forming units (CFUs) at 660 nm. Identification of CagA^+^ *H. pylori* was confirmed using the complete sequence data of the *H. pylori* 16S rRNA gene from GenBank data (sequence ID AP017362) and positive biochemical test reactions for oxidase, catalase, and urease.

### Cell culture and cell-bacteria coculture

The human gastric epithelial cell line GES-1 and intestinal epithelial cell line NCM460 were continuously cultured in Dulbecco’s modified Eagle’s medium (Sigma–Aldrich, USA) containing 10% fetal bovine serum (FBS) (Gibco, New York, USA), 100 IU/mL penicillin G and 100 μg/mL streptomycin (Sigma–Aldrich, USA) in a controlled humidified incubator at 37 °C with 5% CO_2_.

### Reagents, plasmid construction, RNA interference and transfection

The chemical reagents IFN-γ (cat. #PHC4033) was from Gibco (Houston, TX, USA). The recombinant His-CagA protein and the full-length CDX2 plasmid tagged with GFP were purchased from GeneChem Biotechnology Company (Shanghai, China). Short hairpin RNA (shRNA) for CDX2 and its corresponding negative control were also synthesized by GeneChem (Shanghai, China). Transfections were conducted using Lipofectamine 2000 (Invitrogen) according to a previously described protocol [52]. Knockdown efficiencies were determined by immunofluorescence and western blotting, and RNAi sequence with relatively high results was selected for further experiment. The primer information of CDX2, ZO-1, and Claudin-2, as well as targeting sequences of CDX2, are listed in Additional file 1: Table S1.

### Exosome isolation and identification

To prepare exosomes from conditioned media, GES-1 human gastric epithelium cells were cultured with or without CagA^+^ *H. pylori* at a multiplicity of infection of 100 for 2, 6, and 12 h. The conditioned media were then collected from the coculture systems to isolate the exosomes as described [37]. Briefly, cells and debris in conditioned media were eliminated by successive centrifugation (4 °C) at increasing speeds (300 g for 10 min, 2000 g for 20 min, then 10,000 g for 30 min). The supernatant was next transferred to an ultracentrifuge tube and centrifuged at 100,000 g at 4 °C for 70 min twice (Beckman Coulter, Indianapolis, IN). Exosome pellets were resuspended in a small volume containing PBS for further analysis. The protein level in the exosomes was determined by the BCA protein assay (Cat.No.23235; Thermo Fisher, Waltham, MA). The morphologies, size distribution, and biomarkers (HSP70 and CD9) of the exosomes were examined using a transmission electron microscopy (TECNAI G2 Spirit; FEI, Hillsboro, OR), dynamic light scattering with a particle and molecular size analyzer (Zetasizer Nano ZS; Malvern Panalytical, Malvern, Worcestershire, UK), and western blotting, respectively.

### Cell viability and migration assay

Cell proliferation was analyzed using a commercial CCK-8 assay kit (#C0038, Beyotime) and EdU detection kits (RiboBio, Guangzhou, China). The wound scratch assay was performed to evaluate the migration ability. Polarized monolayers of NCM460 cells on transparent supports were washed twice with prewarmed PBS, and wounds were created by scraping the monolayers with a 10 nm pipette tip. Media containing drugs (IFN-γ) was then applied. Twelve random wound points were chosen, and images were taken at time 0 h, 6 h, 12 h, and 24 h. The wound closure area was measured using NIH ImageJ software.

### TEER measurement

To evaluate the barrier integrity of NCM460 cells, the transepithelial electrical resistance (TEER) was measured. The initial TEER was tested before the cells were seeded. Colon cells with and without treatment were seeded in a Transwell membrane insert (12 mm diameter, 0.4 μm pore size, Corning) at a density of 7 × 10^5^ cells/well. Then, 200 μL and 500 μL medium was added to the apical and basal compartments, respectively. Cecropin A (12.5 μg/mL) was added to the apical and basal compartments. The TER values were measured every day by using an ohm-meter fitted with chopstick electrodes (Millipore ESR-2; Burlington, MA, USA). Before each test, the plates were placed at room temperature for 30 min. The TER was calculated by using the following equation: TEER (Ω cm^2^) = (TEER − TER_initial_) × 0.3.

### Luciferase assay

Cells were seeded into 96-well plates and grown to approximately 80% confluency on the second day. Next, the relevant reporter plasmids and the PRL-TK reporter were transiently cotransfected into the cells. After 48 h, firefly luciferase activity and Renilla activity were determined. The ratio of firefly luciferase activity to Renilla activity was regarded as indicating the activity of the gene promoters. Each experiment was performed in triplicate.

### Immunofluorescence staining

Cells were grown on polyethyleneimine-coated coverslips, washed with prewarmed phosphate-buffered saline (PBS), fixed in 4% paraformaldehyde for 15 min, permeabilized with 0.5% Triton X-100 in PBS for 10 min, blocked with 3% bovine serum albumin (BSA) solution for 1 h, and incubated with anti-primary antibodies in 3% BSA at 4 °C overnight (antibody information listed in Additional file 2: Table S2). Cells were then rinsed three times for 5 min with PBS and incubated with individual primary antibodies in 3% BSA at 37 °C for 90 min. Alexa488 or Alexa555 anti-rabbit secondary antibodies (1:100, Invitrogen) were added for 1 h in the dark. Nuclei were counterstained with DAPI (1:1000, sc-3598; Santa Cruz) in PBS at room temperature for 2 min, rinsed with PBS three times for 3 min, and mounted with SlowFade Gold Antifade Reagent (S36942, Life Technologies). Images were obtained from a confocal microscope (TCSSP8, Leica Microsystems) equipped with an acousto-optic beam splitter, a 405-nm laser (for DAPI), an argon laser (488 nm for Alexa 488), and a diode-pumped solid-state (DPSS) laser (561 nm).

### Quantitative real-time RT–PCR and RNA array

Quantitative real-time RT–PCR (qRT–PCR) analyses were conventionally carried out. The primers used for the qPCR of the referenced genes are shown in Additional file 1: Table S1.

### Chronic colitis models of CagA^+^*H. pylori*-infected mice

All animal experiments were performed in accordance with the ARRIVE Guidelines. The study was conducted according to the guidelines of the Declaration of Helsinki, and approved by the Experimental Animal Ethics Committee of the Third Xiangya Hospital of Central South University (The Research Code #LLSC(LA)2018-039, 16 January 2018). Specific-pathogen-free (SPF) male C57BL/6 mice (4 weeks) were cultured in SPF conditions. Forty mice were randomly divided into four groups: 1) mice receiving a standard diet and normal drinking water (control, NC, n = 10); 2) mice administrated *H. pylori* via gavage only (Hp, n = 10); 3) mice receiving PBS via gavage and DSS (DSS, n = 10); and 4) mice receiving CagA^+^ *H. pylori* via gavage and DSS (Hp + DSS, n = 10).

The Hp-infected groups were administered 2.5 × 10^8^ CFU CagA^+^ *H. pylori* by oral gavage once every other day seven times over 13 days to ensure a 100% infection rate. At the same time, the mice in the corresponding control group received PBS by oral gavage as the control. All mice were fasted 12 h before and 4 h after each inoculation. CagA^+^ *H. pylori* colonization in the gastric epithelium was confirmed histopathologically using gastric tissues from the mice after 8 weeks of CagA^+^ *H. pylori* infection with Giemsa and Warthin-Starry silver staining.

For the colitis experiments after 8 weeks of CagA^+^ *H. pylori* infection, the DSS and Hp + DSS groups were subjected to three repeated cycles of 2% DSS (Sigma, St Louis, MO, USA) to induce chronic colitis (7 days/cycle) as instructed [53], and each was separated by 7 days of regular water. During the process, the body weight, disease activity index (DAI) and general status of all mice were observed every three days. Specifically, DAI scoring was measured as described previously (DAI = (Weight loss score + Stool characters score + Hematochezia score)/3) [54]. Mice were sacrificed on the seventh day after returning to drinking water freely. Colon length and the weight of spleen were measured. Colon tissues for histological analyses were dissected and immediately fixed with 4% paraformaldehyde.

The colons were opened longitudinally and briefly cleansed with cold PBS. Tissues were then divided into two equal sections. The proximal parts were fixed in 10% formalin for histological examination while the distal parts were stored at -80 °C for protein extraction. Paraffin-embedded sections of the indicated colons were subjected to H&E staining and then examined by light microscopy. The histological scores were analyzed as described previously [55].

### Protein extraction, immunoprecipitation, and western blot analysis

Protein extracts were obtained from fresh cells and colon tissues with PBS and in RIPA buffer (Thermo Scientific, MA, USA) supplemented with protease inhibitor and phosphatase inhibitor (Roche, Welwyn Garden, Switzerland). Supernatants were eluted in SDS sample buffer and analyzed by SDS–PAGE. For immunoblotting, primary antibodies are indicated in Additional file 2: Table S2. Blots were then developed with ECL western blotting reagents (Pierce Biotechnology, Rockford, IL). Signal intensity was quantified with ImageJ (National Institutes of Health, Bethesda, MD).

### Chromatin immunoprecipitation (ChIP) assay

For ChIP, 1.5 × 10^6^ cells were subjected to a two-step dual cross-linking procedure based on previously described methods [56]. In brief, protein-DNA complexes were immunoprecipitated with anti-CDX2 antibodies followed by q-PCR using a BioRad CFX-96 quantitative thermocycler and SsoFast EvaGreen Low-ROX qPCR SuperMix (BioRad). For qPCR quantification, the following primer pairs are listed in Additional file 1: Table S1. Data were analyzed according to the ΔΔCT method (Applied Biosystems).

### Bioinformatic and statistical analyses

Claudin-2 promoter (− 2000 bp) sequences (H. sapiens) were downloaded from UCSC (http://genome.ucsc.edu/). The specific binding of transcription factors in gene promoters was predicted by the Jasper database (http://jaspar2016.genereg.net/). Statistical analyses were performed using SPSS 19.0 software (version 19.0; SPSS Inc., Chicago, IL). The results are presented as the mean ± S.D., and two-group comparisons were evaluated using Student’s *t test*.

## Supplementary Information

**Additional file 1: Table S1.** Primers and shRNAs used in this study.

**Additional file 2: Table S2.** Antibodies for western blot, immunoprecipitation, and immunofluorescence in this study.

**Additional file 3: Figure S1.** Confirmation of *H.pylori *colonization in gastric mucosa by Giemsa and silver staining in C57BL/6 mice. Stainings in *H. pylori *alone group and *H. pylori+*DSS group indicated that DSS had no effects on *H. pylori* colonization. scale bar, 20µm.

**Additional file 4: Figure S2**. Identification of CagA^-^ *H. pylori *isolated from a gastric ulcer patient’s specimen during gastroscopy. The sequence of the isolated *H. pylori *was compared with the complete sequence of *H. pylori *16s rRNA gene from published GenBank data: sequence ID U00679.1 for CagA^-^ *H. pylori*.

**Additional file 5: Figure S3.** CagA^+^ *H. pylori *infection exacerbates DSS-induced chronic colitis in mice. After one DSS-treatment cycle (7-day 2% DSS and 7-day diluted water), mice without *H. pylori*, with CagA^−^ *H. pylori *infection, and with CagA^+^ *H. pylori *infection were sacrificed. The H&E histological sections of the colon (A), colon lengths (B), and spleen weights (C) were tested in each group. Scale bars, 200 µm (200×) and 100 µm (400×). *p < 0.05, **p < 0.01, ***p < 0.001. Student’s *t test* was used for colon length and spleen weights, and the χ^2^ test was used for histological scores. All data were presented as means ± SD (n = 10).

**Additional file 6: Figure S4.** Cell proliferation and viability assay using EdU and CCK-8. These results indicated the confluence of the NCM460 cell monolayer at the beginning, and the proliferation and survival of colonic cells were not affected by either CagA^+^ exosomes or IFN-γ stimulation.

## Data Availability

Not applicable.

## Abbreviations

*H. pylori*: *Helicobacter pylori*
CagA: Cytotoxin-associated gene A
T4SS: Type IV secretion system
TJ: Tight junction
IBD: Inflammatory bowel disease
CDX2: Caudal related homeodomain transcription 2
ZO: Zonula occludens
GBP1: Guanylate-binding protein 1
TEER: Transepithelial electrical resistance
DSS: Dextran sulphate sodium
