# Supplementary material for: Exosomal CagA from Helicobacter pylori aggravates intestinal epithelium barrier dysfunction in chronic colitis by facilitating Claudin-2 expression
Source: Gut Pathog. 2022 Mar 24;14:13. doi: 10.1186/s13099-022-00486-0 (PMC8944046; doi:10.1186/s13099-022-00486-0)
Supplement: Supplementary file 1 — Additional file 1: Table S1. Primers and shRNAs used in this study. [file 13099_2022_486_MOESM1_ESM.docx]

**Additional file 1: Table S1** Primers and shRNAs used in this study

| **Name** | **Forward/Sense** | **Reverse/Anti-sense** |
| --- | --- | --- |
| CDX2-qPCR | GACGTGAGCATGTACCCTAGC | GCGTAGCCATTCCAGTCCT |
| Claudin-2-qPCR | CGGGACTTCTACTCACCACTG | GGATGATTCCAGCTATCAGGGA |
| ZO-1-qPCR | CAACATACAGTGACGCTTCACA | CACTATTGACGTTTCCCCACTC |
| CagA-qPCR | GATAACAGGCAAGCTTTTGAGG | CTGCAAAAGATTGTTTGGCAGA |
| GAPDH-qPCR | GGAGCGAGATCCCTCCAAAAT | GGCTGTTGTCATACTTCTCATGG |
| β-actin-qPCR | CATGTACGTTGCTATCCAGGC | CTCCTTAATGTCACGCACGAT |
| Claudin-2-qPCR (for CHIP) | CGGGACTTCTACTCACCACTG | GGATGATTCCAGCTATCAGGGA |
| ZO-1-qPCR (for CHIP) | CAACATACAGTGACGCTTCACA | CACTATTGACGTTTCCCCACTC |
| CDX2-shRNA-1 | CCGCAGAGCAAAGGAGAGGAA | TTCCTCTCCTTTGCTCTGCGG |
| CDX2-shRNA-2 | AGACAAATATCGAGTGGTGTA | TACACCACTCGATATTTGTCT |
| CDX2-shRNA-3 | CCGGGAGGACTGGAATGGCTA | TAGCCATTCCAGTCCTCCCGG |
