# Supplementary material for: Exosomal CagA from Helicobacter pylori aggravates intestinal epithelium barrier dysfunction in chronic colitis by facilitating Claudin-2 expression
Source: Gut Pathog. 2022 Mar 24;14:13. doi: 10.1186/s13099-022-00486-0 (PMC8944046; doi:10.1186/s13099-022-00486-0)
Supplement: Supplementary file 2 — Additional file 2: Table S2. Antibodies for western blot, immunoprecipitation, and immunofluorescence in this study. [file 13099_2022_486_MOESM2_ESM.docx]

**Additional file 2: Table S2** Antibodies for western blot, immunoprecipitation, and immunofluorescence in this study

| **Primary antibodies** | **Dilution** | **Company/Catalog #** |
| --- | --- | --- |
| CDX2 | 1:1000 (WB); 1:50 (IP) | Cell Signaling, #3977 |
| Claudin-2 (Human & Mouse) | 1:1000 (WB); 1:100 (IF) | Invitrogen, 12H12 |
| ZO-1 (Human & Mouse) | 1:1000 (WB); 1:200 (IF) | Abcam, ab216880 |
| GBP1 | 1:1000 (WB) | Abcam, ab131255 |
| p-STAT1 | 1:1000 (WB) | Cell Signaling, #8826 |
| Claudin-1 (Mouse) | 1:1000 (WB) | Invitrogen, 2H10D10 |
| Occludin (Mouse) | 1:1000 (WB) | Invitrogen, OC-3F10 |
| CagA (*H.pylori*) | 1:200 (WB) | Santa Cruz, sc-28368 |
| GFP | 1:1000 (WB) | Abcam, ab290 |
| His | 1:500 (WB) | Abcam, ab18184 M |
| Human IgG | 1:150 (IP) | Bioss, #bs-0297P |
| GAPDH | 1:5000 (WB) | Abcam, ab125247 |
| Tubulin | 1:5000 (WB) | Cell Signaling, #12351 |
