# Supplementary figures and images for: Exosomal CagA from Helicobacter pylori aggravates intestinal epithelium barrier dysfunction in chronic colitis by facilitating Claudin-2 expression
Source: Gut Pathog. 2022 Mar 24;14:13. doi: 10.1186/s13099-022-00486-0 (PMC8944046; doi:10.1186/s13099-022-00486-0)

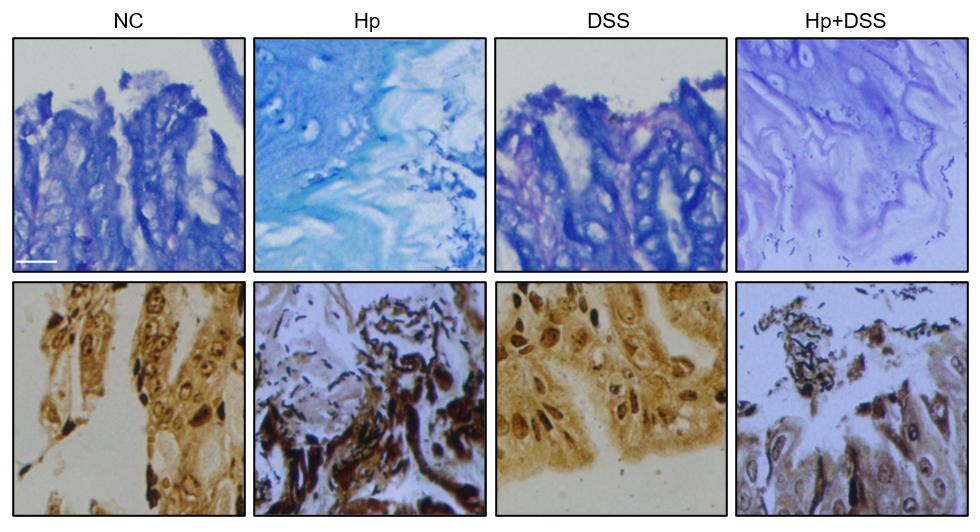

Supplement: Supplementary file 3 — Additional file 3: Figure S1. Confirmation of H.pylori colonization in gastric mucosa by Giemsa and silver staining in C57BL/6 mice. Stainings in H. pylori alone group and H. pylori+DSS group indicated that DSS had no effects on H. pylori colonization. scale bar, 20µm. [file 13099_2022_486_MOESM3_ESM.tif]

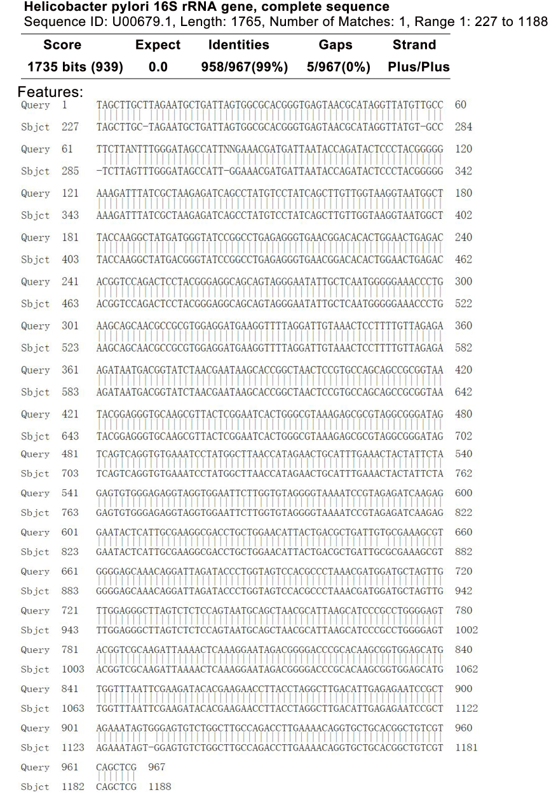

Supplement: Supplementary file 4 — Additional file 4: Figure S2. Identification of CagA- H. pylori isolated from a gastric ulcer patient’s specimen during gastroscopy. The sequence of the isolated H. pylori was compared with the complete sequence of H. pylori 16s rRNA gene from published GenBank data: sequence ID U00679.1 for CagA- H. pylori. [file 13099_2022_486_MOESM4_ESM.tif]

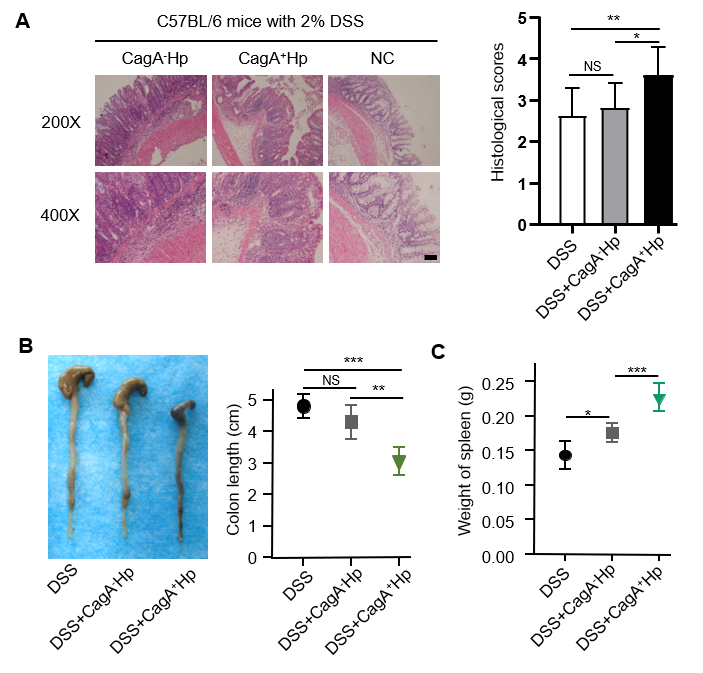

Supplement: Supplementary file 5 — Additional file 5: Figure S3. CagA+ H. pylori infection exacerbates DSS-induced chronic colitis in mice. After one DSS-treatment cycle (7-day 2% DSS and 7-day diluted water), mice without H. pylori, with CagA− H. pylori infection, and with CagA+ H. pylori infection were sacrificed. The H&E histological sections of the colon (A), colon lengths (B), and spleen weights (C) were tested in each group. Scale bars, 200 µm (200×) and 100 µm (400×). *p < 0.05, **p < 0.01, ***p < 0.001. Student’s t test was used for colon length and spleen weights, and the χ2 test was used for histological scores. All data were presented as means ± SD (n = 10). [file 13099_2022_486_MOESM5_ESM.tif]

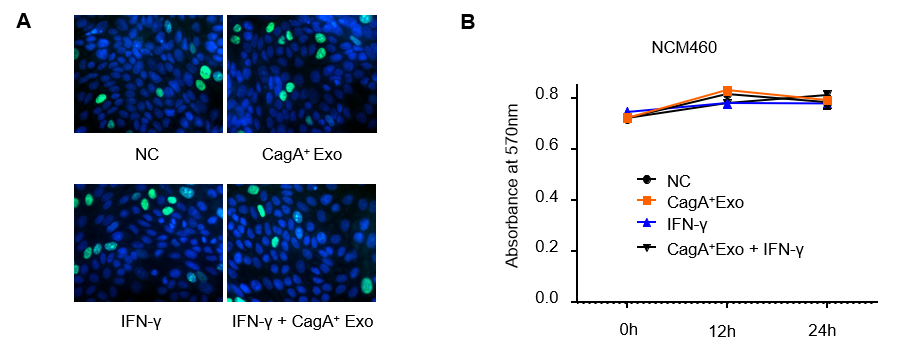

Supplement: Supplementary file 6 — Additional file 6: Figure S4. Cell proliferation and viability assay using EdU and CCK-8. These results indicated the confluence of the NCM460 cell monolayer at the beginning, and the proliferation and survival of colonic cells were not affected by either CagA+ exosomes or IFN-γ stimulation. [file 13099_2022_486_MOESM6_ESM.tif]
